# Supplementary material for: The genome sequence of the beech bark beetle, Taphrorychus bicolor (Herbst, 1793)
Source: Wellcome Open Res. 2024 Apr 23;9:213. [Version 1] doi: 10.12688/wellcomeopenres.21265.1 (PMC11443188; doi:10.12688/wellcomeopenres.21265.1)
Supplement: Peer review history

# Reviewer report: Reviewer response for version 1

**DOI:** 10.21956/wellcomeopenres.23520.r97544

**Published:** 2024-09-30

**Authors:** Daniel Powell

**Affiliations:** 1 University of the Sunshine Coast, Sippy Downs, Queensland, Australia

Dear Editor,

 This manuscript by Telfer et. al., describing the genome assembly and annotation for *Taphrorychus bicolor*, the beech bark beetle is very well presented. The authors provide an informative background supporting the usefulness of these resources and have thoroughly described their results and methodology used in this work. It is critical to build comprehensive resources for these important forest pests to inform and support management strategies for their control.

 I have only one minor comment. The line “The Hi-C data show heterozygous inversions that are not present in the individual from which the DNA was sequenced.“ appears somewhat confusing. If the assembly was made from just the 1 individual, how can these inversions not be present in the individual from which the DNA was sequenced? The authors may wish to revise this statement or to add a clarifying sentence to assist with the readability.

Are sufficient details of methods and materials provided to allow replication by others?

Yes

Is the rationale for creating the dataset(s) clearly described?

Yes

Are the datasets clearly presented in a useable and accessible format?

Yes

Are the protocols appropriate and is the work technically sound?

Yes

Reviewer Expertise:

Ecological genomics

I confirm that I have read this submission and believe that I have an appropriate level of expertise to confirm that it is of an acceptable scientific standard.

# Reviewer report: Reviewer response for version 1

**DOI:** 10.21956/wellcomeopenres.23520.r88296

**Published:** 2024-08-29

**Authors:** Danilo Amaral

**Affiliations:** 1 Universidade Federal do ABC Centro de Ciencias Naturais e Humanas (Ringgold ID: 425753), Santo André, State of São Paulo, Brazil

Dear Editor,

 I'm glad to review the manuscript of Telfer et al. The article presents the genome assembly of the beech bark beetle, *Taphrorychus bicolor*. This species, part of the Curculionidae family, is known for its damaging impact on beech trees, been an important organism to have the genome studied. The genome sequence, spanning 575.2 megabases and including 24,125 protein-coding genes, is scaffolded into 12 chromosomal pseudomolecules. This comprehensive genetic information is crucial for developing efficient and environmentally friendly pest control methods.

 Understanding the genome also aids in identifying its role as a vector for nematodes and other pathogens affecting trees. This reliable data set provides a solid foundation for further research into the beetle's biology and interactions with its environment, which is essential for forest health management.

 The availability of this genome adds to the growing database of sequenced Curculionidae genomes, allowing for comparative studies. These studies can reveal evolutionary adaptations and functional genomics insights within this diverse family. Such knowledge is vital for enhancing pest management strategies and conserving forest ecosystems, ensuring the protection of crucial tree species from damage and disease.

Are sufficient details of methods and materials provided to allow replication by others?

Yes

Is the rationale for creating the dataset(s) clearly described?

Yes

Are the datasets clearly presented in a useable and accessible format?

Yes

Are the protocols appropriate and is the work technically sound?

Yes

Reviewer Expertise:

Bioinformatics

I confirm that I have read this submission and believe that I have an appropriate level of expertise to confirm that it is of an acceptable scientific standard.
